# New Antibiotics for the Treatment of Nosocomial Central Nervous System Infections

**DOI:** 10.3390/antibiotics13010058

**Published:** 2024-01-07

**Authors:** Roland Nau, Jana Seele, Helmut Eiffert

**Affiliations:** 1 Department of Neuropathology, University Medicine Göttingen, Georg-August-University Göttingen, 37075 Göttingen, Germany; 2 Department of Geriatrics, Protestant Hospital Göttingen-Weende, 37075 Göttingen, Germany; 3 Amedes MVZ for Laboratory Medicine, Medical Microbiology and Infectiology, 37077 Göttingen, Germany

**Keywords:** cefiderocol, eravacycline, dalbavancin, avibactam, relebactam, tazobactam, vaborbactam, cerebrospinal fluid, meningitis, ventriculitis

## Abstract

Nosocomial central nervous system (CNS) infections with carbapenem- and colistin-resistant Gram-negative and vancomycin-resistant Gram-positive bacteria are an increasing therapeutic challenge. Here, we review pharmacokinetic and pharmacodynamic data and clinical experiences with new antibiotics administered intravenously for the treatment of CNS infections by multi-resistant bacteria. Cefiderocol, a new siderophore extended-spectrum cephalosporin, pharmacokinetically behaves similar to established cephalosporins and at high doses will probably be a valuable addition in our therapeutic armamentarium for CNS infections. The new glycopeptides dalbavancin, telavancin, and oritavancin are highly bound to plasma proteins. Although effective in animal models of meningitis, it is unlikely that they reach effective cerebrospinal fluid (CSF) concentrations after intravenous administration alone. The β-lactam/β-lactamase inhibitor combinations have the principal problem that both compounds must achieve adequate CSF concentrations. In the commercially available combinations, the dose of the β-lactamase inhibitor tends to be too low to achieve adequate CSF concentrations. The oxazolidinone tedizolid has a broader spectrum but a less suitable pharmacokinetic profile than linezolid. The halogenated tetracycline eravacycline does not reach CSF concentrations sufficient to treat colistin-resistant Gram-negative bacteria with usual intravenous dosing. Generally, treatment of CNS infections should be intravenous, whenever possible, to avoid adverse effects of intraventricular therapy (IVT). An additional IVT can overcome the limited penetration of many new antibiotics into CSF. It should be considered for patients in which the CNS infection responds poorly to systemic antimicrobial therapy alone.

## 1. Introduction

Whereas in most countries the microorganisms causing community-acquired bacterial meningitis are still susceptible to established antibiotics, nosocomial central nervous system (CNS) infections with carbapenem- and colistin-resistant Gram-negative and vancomycin-resistant Gram-positive bacteria are an increasing therapeutic challenge. Since most highly resistant pneumococci and staphylococci are still susceptible to vancomycin and linezolid, and vancomycin-resistant enterococci are usually susceptible to linezolid [1,2], at present, Gram-negative bacteria (in particular *Acinetobacter baumannii* and *Klebsiella* spp.) constitute the greatest threat to patients suffering from nosocomial meningitis and ventriculitis [3,4]. Gram-negative bacteria resistant to fourth-generation cephalosporins, piperacillin/tazobactam, fluoroquinolones, and carbapenems are termed multidrug-resistant (MDR), and, in the case of an additional resistance to polymyxins, they are termed extensively drug-resistant (XDR).

In this review, we will present and discuss pharmacokinetic and pharmacodynamic data and clinical experiences with new antibiotics administered intravenously for the treatment of CNS infections by resistant bacteria. We will not consider less commonly used older antibiotics, which are already established for the treatment of CNS infections either by the intravenous route (e.g., linezolid, aztreonam, or fluoroquinolones) or by combined intrathecal/intravenous therapy (e.g., colistin or tigecycline) because these compounds have been discussed in several reviews (e.g., [5,6,7]), including a recent detailed synopsis of the available evidence in this journal [8].

## 2. Results

### 2.1. β-Lactam Antibiotics

Cefiderocol is a new siderophore cephalosporin exploiting iron transport systems to penetrate bacterial cells and thereby possesses an extended antibacterial spectrum, including carbapenemase- and metallo-β-lactamase-producing Gram-negative bacteria [9]. It is primarily eliminated by the kidneys and possesses an elimination half-life (t_1/2_) of approximately 2–3 h in patients with normal renal function (Table 1). Protein binding in human plasma is approximately 58%. As with other β-lactam antibiotics, cefiderocol is a time-dependent antibiotic [10]. The standard dose in severe infections with normal or slightly decreased renal function (estimated glomerular filtration rate (eGFR) ≥60 mL/min) is 2 g every 8 h. In patients with an eGFR ≥120 mL/min, an increased dose of 2 g every 6 h is recommended [11].

In recent years, several case reports have been published concerning the treatment of CNS infections with multi-resistant pathogens.

In a 41-year-old woman suffering from meningitis after surgery for intracerebral hemorrhage caused by a New Delhi metallo-β-lactamase (NDM)-expressing colistin-sensitive *Pseudomonas aeruginosa* strain receiving 1 g or 1.5 g every 8 h of cefiderocol intravenously (i.v.) plus colistin i.v. and intrathecally, the cerebrospinal fluid (CSF) cefiderocol levels in five samples analyzed were 1.22 mg/L, 1.34 mg/L, 2.39 mg/L, 3.53 mg/L, and 3.90 mg/L. The combination therapy sterilized the CSF, but the patient died on day 50 as a consequence of her underlying cerebral injury. Since the *P. aeruginosa* strain was susceptible both to colistin and cefiderocol, the contribution of each antibiotic to microbiological cure cannot be assessed [12].

In a 77-year-old man, a ventricular drainage placed after surgery for cerebellar arteriovenous malformation was infected by an extensively drug-resistant (XDR) *P. aeruginosa*. Although he had moderate renal impairment (creatinine clearance 44.8 mL/min), he was treated with cefiderocol 2 g every 6 h (duration of each infusion 3 h). Cefiderocol trough and peak serum concentrations collected immediately before and at the end of the 3 h infusion were 105 mg/L (C_min_) and 170 mg/L (C_max_). CSF levels of 13 mg/L were measured 25 min before cefiderocol administration, i.e., at the same time as the serum trough concentrations. The C_min_ CSF/serum ratio was 12.4%, and the patient was microbiologically and clinically cured [13].

A 61-year-old woman with carbapenem-resistant *Acinetobacter baumannii* meningitis was treated with 2 g cefiderocol every 6 to 8 h. The area under the concentration-versus-time curve in CSF (AUC_CSF_) was 146.5 and 118.3 mg·h/L, as determined by the log-linear trapezoidal rule. Penetration into CSF estimated by the ratio AUC_CSF_/AUC_plasma free_ was 68% and 60%. The estimated free plasma and CSF concentrations exceeded the MIC of the isolate for 100% of the dosing interval, and the patient was clinically and microbiologically cured without adverse effects [14].

A 63-year-old man with post-neurosurgical carbapenem-resistant *P. aeruginosa* ventriculitis was treated successfully with cefiderocol 2 g every 6 h administered as 3 h infusions. At steady state, 2 h after the infusion, a peak CSF concentration of 3.6 mg/L, and, immediately before the infusion, a trough CSF concentration of 1.6 mg/L were noted. The serum peak and trough concentrations were 219.2 mg/L and 40.2 mg/L [15].

A patient in her 70s with an external ventricular drain after pituitary surgery developed XDR *P. aeruginosa* ventriculitis. Indeed, 48 h after the start of cefiderocol at 2 g every 6 h, the plasma and CSF trough concentrations were 107.2 and 24.4 mg/L. When the daily dose was reduced to 2 g every 8 h, the trough concentrations in the serum were 39 ± 4.9 mg/L and in CSF 16.8 ± 3.1 mg/L. The CSF became sterile. Since the patient also received daily intraventricular injections of 10 mg colistin and 30 mg amikacin, the contribution of cefiderocol to the microbiological cure is unclear [16].

A 44-year-old man with severe polytrauma following a road traffic accident developed hydrocephalus and received an external ventriculostomy. Infection by a carbapenemase-producing *Klebsiella pneumoniae* was diagnosed and first treated with ceftazidime/avibactam (CZA) plus fosfomycin and linezolid. When this treatment failed and a ceftazidime/avibactam-resistant K. pneumoniae strain was isolated from the CSF, antibiotic treatment was changed to cefiderocol 2 g every 6 h plus trimethoprim/sulfamethoxazole for 14 days. The patient fully recovered. Unfortunately, the cefiderocol concentrations in the CSF were not measured [17]. Since the pathogen isolated was susceptible both to cefiderocol and trimethoprim/sulfamethoxazole, the contributions of the individual compounds to the cure of the patient remain uncertain.

Although complete concentration–time curves in CSF have not been published, preliminary data suggest that cefiderocol (molecular mass 752.2 g/mol) penetrates the blood–CSF and blood–brain barriers slightly less readily than other β-lactam antibiotics with a smaller size (usually <600 g/mol).

Ceftolozane is a cephalosporin with activity against drug-resistant pathogens, including *P. aeruginosa* and *Streptococcus pneumoniae* [18]. It is not cleaved by classical β-lactamases but is not stable against extended-spectrum β-lactamases and carbapenemases [19]. Therefore, it is only available in Europe in combination with tazobactam, and its pharmacokinetics is discussed in the section on β-lactam/β-lactam inhibitor combinations.

### 2.2. Tetracyclines

Eravacycline is a synthetic halogenated tetracycline closely related to tigecycline. It has a broad spectrum of antimicrobial activity, including *Enterobacteriaceae*, *A. baumannii*, *Stenotrophomonas maltophilia*, *S. pneumoniae*, the *S. anginosus* group, *Enterococcus faecalis* and *E. faecium*, *Staphylococcus aureus*, *S. saprophyticus*, *S. epidermidis,* and *S. haemolyticus* [20]. Eravacycline has been suggested for the treatment of carbapenem-resistant *A. baumannii* [9]. The pharmacokinetics of eravacycline was studied in rabbits after single and repeated doses of 0.5–4 mg/kg. Tissues and fluids of the CNS (brain and CSF) and eye (choroid, vitreous, and anterior chamber) had the lowest eravacycline concentrations among all the tissues tested [21]. On day 7, 1 h after the last infusion of 4 mg/kg every 24 h, the CSF and brain concentrations were approximately 0.05 mg/L and 0.17 mg/L [21]. Eravacycline has an apparent volume of distribution of approximately 4 L/kg [22,23] (Table 1). It accumulates in extracerebral tissues, particularly in the kidney, liver, spleen, and lung. For these reasons, and as a consequence of the special conditions at the blood–CSF and blood–brain barriers, the penetration of eravacycline into CSF appears poor [21].

Omadacycline has not been reported for the treatment of CNS infections, and CSF concentrations of this compound have not been published [24].

### 2.3. Glycopeptides and Related Compounds

Dalbavancin is a synthetic highly plasma-protein-bound (approximately 93%) lipoglycopeptide with improved antibacterial potency against Gram-positive organisms and a long plasma half-life of approximately 1 week. It distributes into soft tissues (e.g., skin, bone, and peritoneal space) but reaches only low CSF and brain tissue concentrations [25]. No reports on CSF concentrations or the treatment of patients with CNS infections by dalbavancin have been published.

Telavancin is a semisynthetic lipoglycopeptide derived from vancomycin. It strongly binds to plasma protein (approximately 93%) and has a small volume of distribution and a long elimination half-life [26]. In experimental rabbits, in the absence of meningeal inflammation, the penetration of telavancin into CSF as assessed by AUC_CSF_/AUC_plasma_ was approximately 0.1% and during meningitis approximately 2% [27]. In experimental penicillin-resistant *S. pneumoniae* meningitis, telavancin at a dose of 30 mg/kg of body weight administered i.v. at 0 h and 4 h after intracisternal injection of bacteria was significantly superior to vancomycin (20 mg/kg) combined with ceftriaxone (100 mg/kg). For methicillin-sensitive *S. aureus,* telavancin 2 × 30 mg/kg was only slightly more active than vancomycin 2 × 20 mg/kg (difference not statistically significant) [27]. No reports on the treatment of patients with CNS infections by telavancin have been published yet.

Oritavancin (LY333328) is a new glycopeptide active against Gram-positive bacteria including methicillin-resistant and some vancomycin-resistant strains. Since it possesses an elimination half-life in plasma of 393 h, in adults, 1200 mg of oritavancin is administered as a single dose by intravenous infusion over 3 h. CSF concentrations of 0.013 ± 0.005 mg/L were measured in humans. Based on pharmacokinetic data in plasma, maximum CSF concentrations of 0.01–0.02 mg/L were estimated. These concentrations are below the MICs of many multi-resistant Gram-positive organisms [24], and the dose would have to be increased dramatically to achieve effective CSF levels. To our knowledge, oritavancin has not been used successfully to treat human CNS infections. In a rabbit model of penicillin-sensitive *S. pneumoniae* meningitis, single doses of 2.5 and 10 mg/kg of oritavancin achieved maximum CSF concentrations of 0.54 ± 0.19 and 0.76 ± 0.52 mg/L, respectively, and reduced bacterial concentrations in CSF almost as rapidly as ceftriaxone at 10 mg/kg/h [28]. The CSF-to-serum concentration ratio in most animals was between 1 and 5% [28]. Oritavancin at a dose of 10 mg/kg of body weight/day was also effective in lapine experimental meningitis caused by a cephalosporine-resistant *S. pneumoniae* strain, with CSF levels ranging from 0.09 to 0.33 mg/L [29].

### 2.4. Oxazolidinones

**Figure 1:**
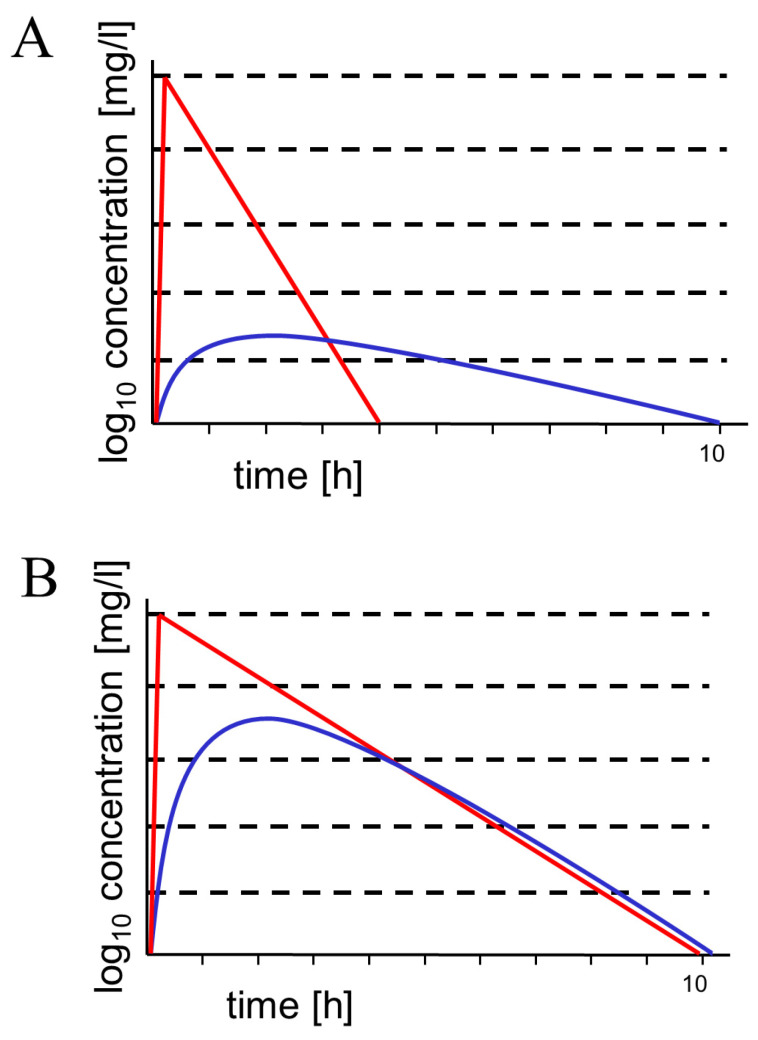
Schematic drawings of concentration–time curves in serum (red) and cerebrospinal fluid (blue). (**A**) hydrophilic molecule with a molecular weight of approx. 300–600 g/moL (such as most β-lactam antibiotics and β-lactamase inhibitors). This type of concentration–time curve has been shown for aztreonam, ceftazidime, meropenem, piperacillin, and tazobactam [5,32,33,34,35] and is also expected for cefiderocol, ceftolozane, and the new β-lactamase inhibitors with an AUC_CSF_/AUC_S_ ratio of 0.02–0.2 depending on molecular weight, hydrophilicity, plasma protein binding, and active transport at the blood–CSF and blood–brain barriers [5]. Since they are smaller than the respective β-lactam, the AUC_CSF_/AUC_S_ ratios of β-lactamase inhibitors are expected to be slightly greater than the AUC_CSF_/AUC_S_ ratios of the respective β-lactams (as shown for piperacillin/tazobactam) [34]. Please note the lagging of the concentration–time curve in CSF behind the respective curve in serum. Because retrograde diffusion across the blood–CSF and blood–brain barriers is small for hydrophilic antibiotics, elimination depends on CSF bulk flow (and to a smaller extent on efflux pumps), leading to long elimination half-lives. (**B**) Moderately lipophilic compound with a molecular weight of approx. 300 g/moL. This type of concentration–time curve represents ideal penetration into CSF (AUC_CSF_/AUC_S_ ratio > 0.8). Linezolid is a prominent example of this relatively rare behavior [31]. Here, the concentration–time curves in serum and CSF almost run in parallel, and the elimination half-lives in serum and CSF are approx. equal. Since tedizolid is highly protein-bound and a ligand of several outward transport systems at the blood–CSF and blood–brain barriers, it is less suitable for the treatment of CNS infections than linezolid.

Tedizolid is an oxazolidinone with a similar antibacterial spectrum as linezolid—some linezolid-resistant Gram-positive cocci are susceptible to tedizolid. Approximately 8 h after a dose of tedizolid 200 mg i.v. every 12 h, CSF concentrations of 0.204 ± 0.006 mg/L were measured, and the CSF penetration based on corresponding unbound plasma concentrations was estimated around 54.8% [24]. In experimental rats in the absence of meningeal inflammation, the mean C_max_ of tedizolid in the CSF was 0.154 mg/L, and the mean penetration ratio of tedizolid into CSF was estimated to be 2.16%. The penetration ratio increased to 3.53% by co-administration of the P-glycoprotein (P-gp) and breast cancer resistance protein (BCRP) inhibitor elacridar, suggesting that P-gp and BCRP are involved in the removal of tedizolid from the CSF [30]. For this reason, intravenous tedizolid appears to be less suitable for the treatment of CNS infections than linezolid, which possesses an ideal pharmacokinetic profile for this indication [31] (Figure 1B).

### 2.5. Aminoglycosides

Plazomicin is a new semisynthetic aminoglycoside antibiotic structurally derived from sisomicin. It has not been reported to be used for the treatment of CNS infections.

### 2.6. β-Lactam Antibiotic/β-Lactamase Inhibitor Combinations

All novel β-lactam antibiotics/β-lactamase inhibitors have similar pharmacokinetic properties, i.e., hydrophilicity, low binding to plasma proteins, low molecular mass, a small volume of distribution, and a predominantly renal clearance [36], suggesting moderate entry into the central nervous compartments with mild or no impairment of the blood–CSF and blood–brain barriers (Table 1). The use of β-lactam/β-lactamase inhibitor combinations for the treatment of CNS infections comprises a principal problem: for in vitro determination of susceptibility, in particular for the determination of minimal inhibitory concentrations (MICs) in broth, a fixed β-lactamase inhibitor concentration is used, whereas the concentration of the β-lactam antibiotic is titrated. The fixed β-lactamase inhibitor concentrations recommended by the European Committee on Antimicrobial Susceptibility Testing (EUCAST) are 4 mg/L (avibactam, relebactam, or tazobactam) or 8 mg/L (vaborbactam) [37,38]. These β-lactamase inhibitor concentrations used for in vitro MIC determinations are higher than the β-lactamase inhibitor concentrations reached in CSF [34,39]. Therefore, the susceptibility testing of β-lactam/β-lactamase inhibitor combinations for pathogens causing CNS infections is hampered with a systematic error. Since the penetration expressed as the ratios of the AUCs in CSF and plasma is only moderately increased for β-lactamase inhibitors compared to β-lactams [34], in piperacillin/tazobactam with a fixed β-lactam/β-lactamase inhibitor combination ratio of 8, tazobactam is probably underdosed. Fortunately, in new commercially available β-lactam/β-lactamase inhibitor combinations, the dose of the β-lactam is only 1 (meropenem/vaborbactam), 2 (ceftolozane/tazobactam, imipenem/relebactam), 3 (aztreonam/avibactam), or 4 times (ceftazidim/avibactam) greater than the dose of the β-lactamase inhibitor.

Ceftolozane/tazobactam is a novel β-lactam/β-lactamase inhibitor combination active against multidrug-resistant *P. aeruginosa* and some other ESBL-producing Gram-negative bacteria [40]. Ceftolozane/tazobactam has been successfully used to treat CNS infections caused by *P. aeruginosa* unresponsive to meropenem [41,42,43]. During a continuous infusion of 6 g ceftolozane and 3 g tazobactam over 24 h for 6 days in a patient with strong meningeal inflammation, the ceftolozane CSF concentrations were 38.8 (19 h after start of the infusion) and 55.2 mg/L (6 d after start of the infusion). A ceftolozane serum concentration of 46.6 mg/L was measured 15 h after the start of the infusion. Assuming steady state conditions already on day 1, this would result in a CSF penetration of 83%. The tazobactam concentrations were not determined [43]. After a single dose of 2 g ceftolozane/1 g tazobactam infused i.v. over 1 h in 10 patients with mild or without meningeal inflammation, the median (25th/75th percentile) areas under the unbound concentration–time curve (fAUC_0-infinity_) of ceftolozane in the CSF were 30 (19/128) h∙mg/L and for tazobactam 5.6 (2/24) h∙mg/L. Based on the CSF-to-plasma AUC ratio, the CSF penetration was 0.2 ± 0.2 and 0.2 ± 0.26 for ceftolozane and tazobactam, respectively. The interpatient variability was high and depended on the degree of inflammation in the individual patient. For all except one, the measured CSF tazobactam concentrations were below 4 mg/L [39]. During continuous treatment of a 39-year-old man suffering from meningitis caused by multidrug-resistant *P. aeruginosa* with ceftolozane/tazobactam (2 g + 1 g), ceftolozane CSF concentrations of 4.13 and 6.98 mg/L and tazobactam CSF concentrations of <0.4 and 0.82 mg/L were measured [44]. Despite a dose of 1 g every 8 h, most tazobactam CSF concentrations were below the fixed concentration of 4 mg/L used for routine in vitro MIC testing, indicating that the susceptibility testing of pathogens causing CSF infection with a fixed tazobactam concentration of 4 mg/L is unreliable [39,44].

Aztreonam–avibactam is a promising combination for the therapy of infections by Gram-negative bacteria producing metallo-β-lactamases (MBL) [45]. Two patients suffering from post-surgical CNS infections by XDR *P. aeruginosa* were successfully treated with aztreonam and avibactam plus ceftazidime. In these patients, either ceftolozane/tazobactam had failed or colistin had caused acute kidney injury [42]. Aztreonam penetrates the CSF more readily in patients with inflamed meninges than in the absence of meningeal inflammation [32,46]. With uninflamed meninges, 4.7 h after an i.v. infusion of 2 g, maximum CSF concentrations of 1.03 ± 0.20 (0.83–1.22) mg/L were measured. Based on the ratio AUC_CSF_/AUC_S_ from 0.5 to 8 h, the CSF penetration of aztreonam in the absence of meningeal inflammation was estimated to be 0.015. The concentration-versus-time curves in CSF and serum constructed from individual measurements showed the typical course of a hydrophilic drug with a molecular weight around 500 g/moL, i.e., a lag of the CSF curve behind the serum curve and a considerably slower elimination from CSF than from serum (Figure 1) [32]. For this reason, the use of the AUC_0-8h_ instead of AUC_0h-∞_ from CSF and serum moderately underestimated the true CSF penetration [5]. In the presence of meningeal inflammation, after the same intravenous dose, CSF maxima of 3.22 ± 2.99 (1.01–6.63) mg/L were noted 4.2 h after infusion [32].

Ceftazidime–avibactam is active against enterobacteria producing extended spectrum β-lactamases (ESBL), *K. pneumoniae* carbapenemase (KPC), AmpC type (class C), and some class D β-lactamases and against carbapenem-resistant *P. aeruginosa* [40,42]. The CSF penetration of ceftazidime is well-characterized: after a dose of 3 g i.v., the maximum CSF concentrations were reached 1 to 13 h (median, 5.5 h) after the end of the ceftazidime infusion and ranged from 0.73 to 2.80 mg/liter (median, 1.56 mg/liter). As expected for hydrophilic compounds, the elimination half-lives in CSF were longer (3.13 to 18.1 h (median, 10.7 h)) than in serum (2.02 to 5.24 h (median, 3.74 h)). The AUC_CSF_/AUC_S_ ratio ranged from 0.027 to 0.123 (median, 0.054) and was similar to the AUC ratio of β-lactam antibiotics with a similar molecular mass and binding to plasma proteins [5,33]. Therapy with ceftazidime 2 g/avibactam 0.5 g administered as an infusion over 2 h every 8 h in meningitis resulted in avibactam concentrations in CSF of approximately 4 mg/L 130–184 min after the start of the infusion. Yasmin and co-workers estimated that avibactam CSF concentrations ≥1–2.5 mg/L were maintained for approximately 50% of the dosing interval [47]. In a successfully treated 4-year-old girl with post-neurosurgical meningitis and abscess caused by extended-spectrum β-lactamase-producing *E. coli*, ceftazidime (15.6, 7.1, and 3.5 mg/L) and avibactam concentrations (4.0, 2.1, and 1.2 mg/L) were determined in CSF samples drawn 3, 5, and 7 h after the infusion of the 15th dose (2 g ceftazidime, 0.5 g avibactam every 8 h). The corresponding serum concentrations at 3 and 5 h after the infusion were 57.0 and 25.8 mg/L (ceftazidime) and 11.3 and 4.5 mg/L (avibactam) [48].

Meropenem/vaborbactam is active against ESBL-, KPC-, or AmpC-producing enterobacteria [40]. Apparently, there are no data concerning the entry of vaborbactam into the central nervous compartments or any reports on the use of meropenem/vaborbactam in CNS infections. Conversely, meropenem is well-established with respect to its kinetics in CSF [35] and its use for bacterial meningitis at a recommended standard dose of 3 × 2 g/day [49].

Compared to imipenem/cilastatin, imipenem/relebactam (plus cilastatin) is active against ESBL-, KPC-, PDC-, and AmpC-producing enterobacteria and also has some additional activity against *P. aeruginosa*. It is inactive against metallo-β-lactamases and class-D-carbapenemases [50]. To our knowledge, this combination has not been studied with respect to CSF penetration and has not been used for CNS infections. As a consequence of the high incidence of seizures (up to 33%) in patients not having seizures prior to the administration of imipenem, meropenem is preferentially used compared to imipenem/cilastatin for the treatment of bacterial meningitis [49,51]. For this reason, imipenem/relebactam (plus cilastatin) will probably not play a major role in the treatment of CNS infections.

## 3. Methods

We searched PubMed and PMC for the following terms: meningitis or ventriculitis or brain abscess or cerebrospinal or CSF and name of the respective antibiotic/antibiotic combination.

## 4. Discussion

The entry of an antibiotic into CSF is determined by the properties of the drug and of the host: with small molecules (molecular weight 100–1000 g/moL), the most important physicochemical drug property for the entry into the CSF is lipophilicity at pH 7.4. In this respect, the rule established by Ernst Overton in 1900 studying the entry of anilin dyes through the cell membranes of living plant cells [52] is still valid. The other strong determinants of entry into the CSF are molecular size and binding to plasma proteins. The molecular masses of the compounds studied here are similar and were below 1000 g/moL with the exception of the new glycopeptides. Therefore, with respect to antibiotics, molecular weight accounts for less of the variation in drug entry into the CSF than lipophilicity. For large molecules, e.g., proteins, molecular weight is a strong determinant of drug entry into the CSF [53,54]. Drug binding to plasma proteins strongly influences drug entry into the CSF because, in general, only the free drug fraction is ready to penetrate [5,8].

Active transport can have a moderate influence on the CSF concentrations of antibiotics. For the following compounds studied in this review, active outward transport mechanisms across the blood–CSF and blood–brain barriers have been described. The organic anion transporter 3 (OAT3) located in the choroid plexus and at the blood–brain barrier is able to remove penicillin G, cephalotin, and similar antibiotics from the central nervous compartments. However, ceftriaxone and imipenem appear not to be ligands to this outward transport system [55]. For other newer β-lactams and β-lactamase inhibitors, the contribution of the organic anion transporter 1 (OAT1) or OAT3 at the blood–CSF and blood–brain barriers to the restriction of drug entry into CSF has not been studied. Data from renal drug transport suggest that meropenem, piperacillin, avibactam, and tazobactam are weak ligands of OAT1/3 [56,57,58]. In vitro, relebactam was shown to be a substrate of OAT3, the organic anion transporter 4 (OAT4), and of the multidrug and toxin extrusion (MATE) proteins MATE1 and MATE2K [59]. It should be noted that, in experimental animals, in the absence of meningeal inflammation, probenecid increased the CSF-to-serum concentration ratio of the strong OAT3 ligand penicillin G during continuous i.v. infusion by 2 to 3 times, and this effect tended to be weaker during meningitis [60]. For this reason, the detrimental role of active drug transport at the blood–brain and blood–CSF barriers—with the exception of some compounds such as cephalotine, which are not used for the treatment of CNS infections—should not be overestimated.

Host variables independent of the physicochemical properties of the drug are (a) the patient’s age, (b) volume of CSF and of other intracranial compartments, (c) CSF flow, which may be influenced by underlying diseases affecting the CSF production rate and by drugs, (d) plasma albumin influencing the protein binding of the drug, and (e) polymorphisms of genes encoding the transport proteins. The CSF concentrations of drugs also depend on the site of measurement; i.e., after intravenous infusion (or oral ingestion), generally, there is a rostrocaudal concentration gradient: drug concentrations are lowest in ventricular, intermediate in cisternal, and highest in lumbar CSF. The size of this concentration gradient does not only depend on CSF flow but also on anatomical conditions, such as the person’s height, width of the spinal canal, and obstructions of the CSF flow by diseases such as spinal disc herniations, blood clots, or tumors [54]. Because of the great heterogeneity of the conditions of the host, it is very difficult to predict CSF concentrations based on physicochemical drug properties and pharmacokinetic data in the systemic circulation in clinical practice [54]. Antibiotic concentrations measured in ventricular CSF in the absence of meningeal inflammation represent the lowest concentrations a clinician must be aware of when treating nosocomial ventriculitis accompanied by mild inflammation.

In the absence of strong meningeal inflammation, all the compounds presented here have difficulties in attaining adequate CSF concentrations (Figure 1). The easiest way to increase CSF concentrations in this condition is to increase the intravenous daily dose. This has been successfully pursued with older antibiotics: the daily dose of cefotaxime has been increased up to 24 g, and the daily dose of meropenem up to 15 g to achieve bactericidal CSF levels in meningitis caused by bacteria with a reduced susceptibility to antibiotics [61,62,63]. With the compounds discussed here, no reports on increasing the daily doses strongly beyond the doses recommended by the manufacturers are available. As a consequence of the increasing resistance of the pathogens causing nosocomial meningitis, case reports on off-label use with increased doses will probably soon appear.

Intrathecal therapy is not an option for β-lactam antibiotics and β-lactamase inhibitors because of their pro-convulsive properties [6,64]. Since vancomycin is an established drug for intrathecal therapy and tigecycline has been administered intrathecally with tolerable adverse effects, intrathecal in addition to intravenous therapy may be an option of last resort for new glycopeptides and eravacycline.

## 5. Conclusions

Among the new antibiotics administered intravenously with a spectrum of interest for the treatment of CNS infections, the pharmacokinetics of cefiderocol in CSF appears to be similar to the kinetics of other β-lactam antibiotics. Provided that high doses are tolerated (with normal renal function ≥6 g/day), adequate CSF concentrations to successfully treat carbapenem-resistant bacteria can be assumed. Therefore, this drug appears to be the first choice for the treatment of CNS infections caused by multi-resistant Gram-negative bacteria.

Because of the problems of the susceptibility testing of β-lactam/β-lactamase inhibitor combinations for pathogens causing CNS infections, the use of β-lactam/β-lactamase inhibitor combinations for the treatment of CNS infections comprises a principal problem: the use of β-lactamase inhibitor concentrations for in vitro susceptibility testing, which will not be reached in human CSF with conventional dosing. Nevertheless, these combinations administered intravenously are therapeutic options of last resort for cefiderocol-resistant bacteria.

Generally, treatment of CNS infections should be intravenous, whenever possible, to avoid adverse effects of intraventricular therapy. Since many antibiotics do not cross the blood–CSF and blood–brain barriers readily, it can be difficult to treat intracranial infections caused by multi-resistant bacteria by intravenous administration alone [65]. Intraventricular in addition to intravenous therapy should be considered for patients in which the CNS infection responds poorly to systemic antimicrobial therapy [6,7,66]. Eravacycline and new glycopeptides have an antimicrobial spectrum that is also of interest for the treatment of CNS infections. Because of their relatively large molecular size (glycopeptides) and strong binding to several tissues (eravacycline), these antibiotics reach low CSF concentrations after intravenous administration. Therefore, they may be unsuitable for the intravenous treatment of CNS infections alone but may be suitable for combined intravenous/intraventricular therapy. β-lactam antibiotics and β-lactamase inhibitors should not be administered intrathecally because of their pro-convulsive properties.

**Table 1:** Pharmacokinetic properties of novel β-lactams and/or β-lactams/β-lactamase inhibitor combinations and the tetracycline eravacycline (taken from [36], unless specified otherwise). In critically ill patients, strong deviations of these parameters can be encountered [67].

Drug	Molecular Mass	Hydrophilicity XLOGP3-AA	V_d_ [l]	t_1/2_ [h]	Protein Binding [%]	Renal CL [%]
Cefiderocol	752.2	1	13.5/26.6	2–3	40–60	90–98
Aztreonam–avibactam	435.4/265.3	0.3/−1.8	11.2 */22.2	1.7–2 */1.5–2 ^§^	56 */6–8 ^§^	>90 */>90 ^§^
Ceftazidime–avibactam	546.6/265.3	−0.21/−1.8	17.0/22.2	1.5–2.7/1.5–2 ^§^	7–10/6–8 ^§^	72–87/>90 ^§^
Ceftolozane–tazobactam	666.7/300.3	−3.2/−2	13.5/18.2	3.1/2.3 ^§^	16–30/30 ^§^	62–84/80 ^§^
Imipenem–relebactam	299.4/348.4	−0.7/−3.6	19.0/24.3	1.2/1.2 ^§^	20–22/22 ^§^	52–92/>90 ^§^
Meropenem–vaborbactam	383.5/297.1	−2.4/nd	18.6/20.2	2.3/1.6 ^§^	2–33/33 ^§^	74/75–95 ^§^
Eravacycline	558.6	0.24 #	321	24	80–90	34

V_d_—volume of distribution; t_1/2_—half-life; CL—clearance. XLOGP3-AA is an atom-additive method that calculates log P by adding up contributions from each atom in the given molecule [68]. The XLOGP3-AA values of this table stem from https://pubchem.ncbi.nlm.nih.gov (cefiderocol: https://pubchem.ncbi.nlm.nih.gov/compound/Cefiderocol; aztreonam: https://pubchem.ncbi.nlm.nih.gov/compound/aztreonam; ceftolozane: https://pubchem.ncbi.nlm.nih.gov/compound/Ceftolozane; imipenem: https://pubchem.ncbi.nlm.nih.gov/compound/imipenem; https://pubchem.ncbi.nlm.nih.gov/compound/meropenem; avibactam: https://pubchem.ncbi.nlm.nih.gov/compound/Avibactam; tazobactam: https://pubchem.ncbi.nlm.nih.gov/compound/Tazobactam; relebactam: https://pubchem.ncbi.nlm.nih.gov/compound/Relebactam; vaborbactam: https://pubchem.ncbi.nlm.nih.gov/compound/Vaborbactam; retrieved 24 December 2023) or Wardecki et al. [69] (ceftazidime). nd—not determined. #no XLOGP3-AA estimate available; log P estimated by ALOGPS, Table S4 [23]. * Data for aztreonam provided by Mattie [46] assuming a body weight of 70 kg. ^§^ Data provided by Barbier et al. [67].

## Data Availability

Not applicable. Only previously published data have been used in this manuscript.
